# A multi-step approach to managing missing data in time and patient variant electronic health records

**DOI:** 10.1186/s13104-022-05911-w

**Published:** 2022-02-17

**Authors:** Nina Cesare, Lawrence P. O. Were

**Affiliations:** 1 Boston University School of Public Health, Biostatistics and Epidemiology Data Analytics Center, Boston, MA USA; 2 Department of Health Sciences & School of Public Health, Department of Global Health, Boston University Sargent College, Boston, MA USA

**Keywords:** Electronic medical records, HIV, Imputation, Big data

## Abstract

**Objective:**

Electronic health records (EHR) hold promise for conducting large-scale analyses linking individual characteristics to health outcomes. However, these data often contain a large number of missing values at both the patient and visit level due to variation in data collection across facilities, providers, and clinical need. This study proposes a stepwise framework for imputing missing values within a visit-level EHR dataset that combines informative missingness and conditional imputation in a scalable manner that may be parallelized for efficiency.

**Results:**

For this study we use a subset of data from AMPATH representing information from 530,812 clinic visits from 16,316 Human Immunodeficiency Virus (HIV) positive women across Western Kenya who have given birth. We apply this process to a set of 84 clinical, social and economic variables and are able to impute values for 84.6% of variables with missing data with an average reduction in missing data of approximately 35.6%. We validate the use of this imputed dataset by predicting National Hospital Insurance Fund (NHIF) enrollment with 94.8% accuracy.

## Introduction

Electronic Health Records (EHRs) are systematized sources of patient data that medical providers collect and store using digital tools. They have the potential to improve patient care by providing access to rich, longitudinal, patient-level data that may be used to advance precision medicine and lead to more personalized care [1–5]. In addition to facilitating more customized care, EMRs can be used to build machine learning models and generate new insights regarding patient behavior, biology and health outcomes [6–8].

One of the primary challenges posed by EHRs is that they often contain large amounts of missing data [2, 4, 9]. Practitioners entering data for a clinical-encounter may only elect to enter fields relevant to the patient’s clinical needs at that time, and data may be aggregated across sites that have varying standards for record-keeping [10]. By proposing a scalable, stepwise system for imputing considerable volumes of missing values we hope to make EMR data more accessible researchers interested in leveraging the *big data* aspects of these records, and promote collaboration between medical researchers and data scientists.

### Handling missing data in electronic health records

Approaches toward managing missing EHR data vary. In summarizing the use of EHR data to develop risk prediction models, Goldstein et al. [9] found that only 58 of the 90 studies evaluated addressed missing data prior to analysis. The simplest approaches toward managing missing values involve selecting subsets of the data that contain complete information [11, 12], and using stratified mean imputation used to fill-in missing values [13]. Others have designed functions to interpolate longitudinal variables with limited individual-level variability that are typically not dependent on other covariates [14]. This approach is applicable only to continuous measures. Few studies using EHR utilize ‘informative observations’ where the presence of a variable is meaningful for associated, possibly missing values [9].

Simpler approaches toward EHR imputation must consider whether missing values are missing completely at random (MCAR), missing at random (MAR), or missing not at random (MNAR) [14]. Conditional imputation methods may be used to account for these dependencies, most effectively if missing data are MAR [10, 12, 15]. While they may improve completeness and predictive precision, these methods may be computationally intensive when applied to large-scale EHR data with significant amounts of missing values.

### Research objectives

**Fig. 1:**
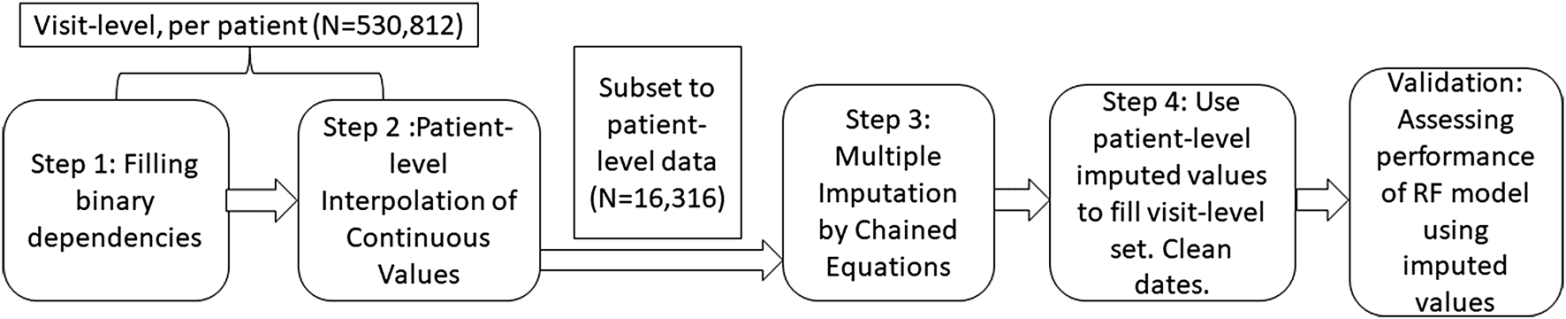
Visual representation of workflow, noting unit of analysis shifts

Our goal in this study is to design and utilize a scalable, multi-step approach toward imputing missing values in EHR data (Fig. 1). Our approach recognizes that EHR data is both patient- and time-variant, but that collection methods do not produce data that closely resembles repeated measures from a random population [16]. To assess results, we measure differences in response coverage for each variable, and feed our results into a decision tree classifier designed to predict NHIF enrollment. The ability to accurately predict National Hospital Insurance Fund (NHIF) enrollment serves as validation for the utility of using predicted values in research.

### Data used

For this study we use data from AMPATH—a robust EHR system containing health records for over 150,000 HIV + individuals across Western Kenya. Past research has focused on cohort studies of AMPATH data, selecting measures and observations that minimize the missingness of these data [17]. We specifically focus on a subset of these data that contain longitudinal information from 530,812 clinic visits for 16,316 HIV positive women who have given birth. Clinic visits span from December 19th, 2001 to September 2nd, 2013. The average number of clinic visits per patient is 32 with a standard deviation of 23. The maximum number of visits across this dataset is 162.

This analysis focuses on a set of 84 variables of interest to researchers studying HIV diagnosis and maternal health outcomes among HIV positive Kenyan women [17, 18]. Approximately 93% of these variables contain missing values across visits. Among these, 42% are missing more than half of possible recorded values. Variables included access factors such as antiretroviral (ARV) medication regimen, clinical HIV wellness information such as CD4 count and viral load, diagnosis of respiratory illness, delivery information, social and economic background information about the mother, and NHIF enrollment.

## Main text

### Methods

#### Step 1: filling binary variable dependencies in visit-level data

Missing values in the dataset may be non-applicable, or they may represent “no” values depending on the interface available or the decisions of the individual entering data. Using domain expertise, we identify dependencies between variables and develop a function that uses these dependencies to fill missing “no” values within binary variables. This step is performed using visit level data for each patient. Not only does this cleaning step improve data coverage, it provides variance that may be helpful when filling in other values. Figure 1 summarizes this and all subsequent analytic steps.

#### Step 2: patient-level interpolation of continuous values

For continuous, individual-level variables that are missing instances of reporting throughout the dataset, we rely on linear interpolation. Examples of variables imputed by this step include body mass index (BMI), systolic and diastolic blood pressure, arterial oxygen saturation (Sa02). We elect to build linear models using observed data and predict values for gaps based on this relationship. For this step, we utilize visit level data for each patient. Because this step relies on individuals’ data, it can be completed in parallel and is highly scalable for larger datasets.

#### Step 3: multiple imputation by chained equations for patient-level data

Using the dataset that has been partially filled based on the previous two steps, we use multiple imputation by chained equations (MICE) to fill the remaining values. First, due the challenge of accounting for temporal variation in this dataset, we convert the data from visit-level to patient level prior to MICE imputation by randomly selecting one observation to represent an individual. MICE is a conditional imputation approach that has been shown to be effective for imputing EHR data with low error [10, 19]. This imputation process leverages five distinct steps. First, it creates multiple copies of the dataset and replaces missing values with randomly selected, temporary ‘placeholder’ variables. Separate regression models are used to impute missing values across data copies for each variable. These predictions are then pooled, creating a set number of candidate, imputed datasets from which we randomly select a value. Given that the majority of responses are categorical and variation is minimal, we do not anticipate random selection to change the structure of the data. To complete this step, we use the package *mice* in R [20], and specify the use of classification and regression trees with five data copies to fill values.

#### Step 4: filling visit-level dataset and cleaning date variables

To create values that represent individuals and reduce the possible impact of outliers, we randomly select one row per patient. Finally, because we note that MICE incorrectly fills in date values for two variables—delivery date and CD4 count date—we elect to complete our data generation process by carrying the last available date forward for each patient.

#### Validation using random forest modeling

We validate imputed values by building a random forest model designed to predict enrollment in the NHIF. This measure is both patient- and time-variant, and it is present for 107,566 visits within the dataset. We randomly select 80% of the dataset to use as a training set, and 20% to use as a test or validation set. The random forest model includes fourteen measures expected to correlate with NHIF enrollment, all of which were included in the imputation process. We then measure the performance of this classifier by comparing the accuracy and F1-scores of the predicted and observed test set enrollment values (Table 1).

### Results

#### Filling binary variable dependencies in visit-level data

The majority of dependency-filled variables relate to ARV medication regimens. We assume that if individuals report: (a) being on ARV but; (b) have no reported change in ARV regimen, then we mark all subsequent change behaviors as values as “no” (Table 1). This approach led to an average 72.4% increase in the number of visit-level values filled.

**Table 1:** Variables imputed using value dependencies

Dependency 1	Dependency 2	Variable of interest	Original variation		Imputed variation	
			No	Yes	No	Yes
On ARV	NA	Change in ARV regimen	0	1375	386,984	1375
On ARV	Change in ARV regimen	ARV stop: Completed T-pMTCT	0	2588	384,488	2588
On ARV	Change in ARV regimen	ARV stop/change due to regiment failure	0	352	386,937	352
On ARV	Change in ARV regimen	ARV stop/change due to toxicity	0	1696	385,509	1696
On ARV	Change in ARV regimen	ARV stop/change due to weight change	0	10	386,974	10
On ARV	Change in ARV regimen	ARV stop/change due to other reason	0	2002	385,266	2002
On ARV	Change in ARV regimen	ARV stop/change due to new TB	0	43	386,942	43
On ARV	Change in ARV regimen	ARV stop/change due to non-adherence	0	358	386,637	358
On ARV	Change in ARV regimen	ARV stop/change due to out of stock	0	63	386,927	63

#### Step 2: patient-level interpolation of continuous values

Using the parameters identified, we interpolated values for 33 variables in our dataset. These variables represent variables that are numeric and have at least ten unique values across the total dataset. The majority of these values are continuous values with variance that depends only on the individual and not on the cohort or clinic. Because we have a limited number of observations for many individuals, this limits our degrees of freedom and inhibits the inclusion of control variables and smoothing parameters.

**Fig. 2:**
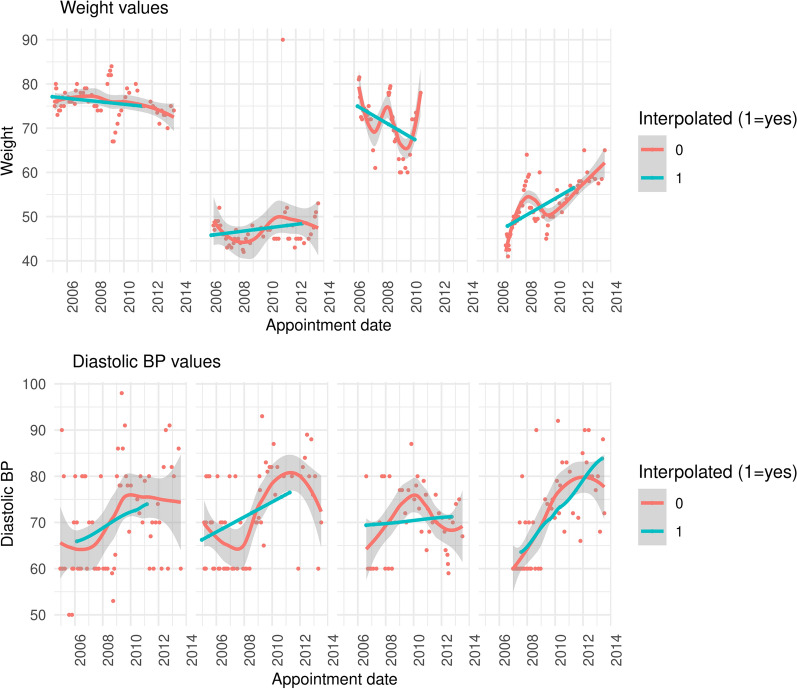
Patient weight and Diastolic BP over time for several patients among a random subset of 100 patients. Red indicates observed weight, and blue indicates imputed weight

Variables addressed using this technique include continuous measures such as: body weight, CD4 count, and diastolic/systolic blood pressure. Using these four variables as an illustration, we find that within the visit-level dataset, approximately 10.9% and 11.0% of entries are missing for systolic and diastolic blood pressure, respectively, 10.1% are missing for body weight, and 84.9% are missing for CD4 count. Interpolating values per patient drops the number of missing visit-level values to zero for all variables. Figure 2 illustrates that filling visit-level data gaps with time-based linear interpolation produces results that follow patterns similar to those observed for patients.

#### Steps 3 and 4: MICE for patient level data, filling and cleaning visit-level dataset

**Fig. 3:**
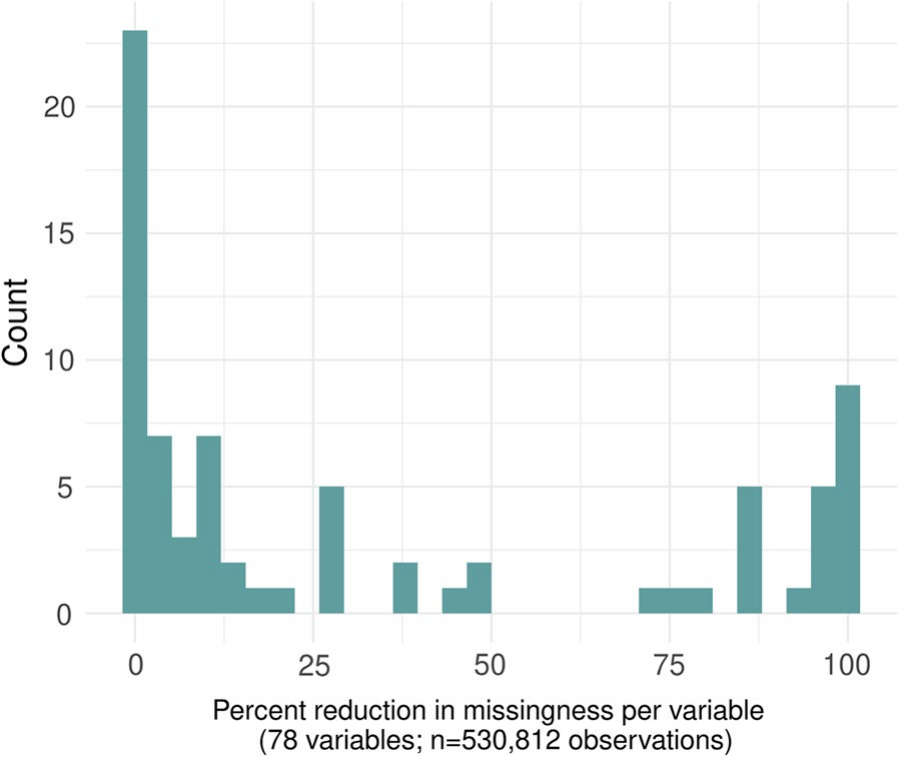
Percent reduction in missing values pre- and post-imputation among focal variables

We next reduce our dataset from visit level to patient-level, apply MICE imputation, and use generated values to fill gaps in the visit-level dataset. Comparing the original dataset to the final, imputed dataset, we note that 78 focal variables required imputation. At least some missing values were filled for 66 (84.6%) of variables. Only 12 variables saw no reduction in missing values. Figure 3 displays the change in percent missing per variable pre- and post-imputation. We note that 20 variables saw a percent reduction in missing values of 80% or more. In terms of processing time, we completed all cleaning and imputation steps within 6 hours by parallelizing each component.

#### Validation

We validate our data by predicting visit-level NHIF enrollment using a random forest algorithm. Within our data, an estimated 7% percent of women are enrolled, which is in line with national estimates of approximately 11% [21]. Our model uses the following measures as predictors: pregnancy outcomes and location/delivery help; the state of illness as defined by WHO weight loss state, respiratory infection, viral load, and ARV medication regimen; socioeconomic status as measured by educational attainment and, and select background characteristics including age, age at first pregnancy, and number of children under 18 months of age. We find that despite class imbalance, our classifier performs well and achieves an accuracy of 94.8% and an F1 score of 0.587 (Appendix: Table 2).

Furthermore, indications of component variable significance (Appendix: Table 3), illustrate that indicators of illness progression such as BMI and later WHO weight loss stages are significant predictors of NHIF enrollment. Education is also a key predictor; a bivariate analysis indicates that those with recorded insurance have an average of 9 years of education versus 7.5 years of education among those who do not (t = 112.54, p < 0.001). These factors correspond with existing research addressing determinants of healthcare access among rural Kenyan women [18, 22].

### Discussion

The scale of EHR data offer numerous advantages for health researchers. The primary challenge of using these data, however, is that they typically contain a number of missing values [2, 4, 9]. By leveraging this step-wise cleaning and imputation process, we are able to fill values for 84.6% of selected variables with initial non-zero missingness on a dataset that contains information on 530,812 visits for 16,316 patients. A predictive model utilizing these data is highly accurate and validates what is known about health insurance enrollment among rural Kenyan women [17, 18, 21].

One of the goals underlying this work is to encourage researchers across disciplines to leverage the ‘big data’ aspects of EHR [4, 5]. Rather than reduce their dataset to a handful of predictors or a single cohort of observations, researchers may use the full dataset and engage in more data-driven, predictive model building and gain new insights into the association between healthcare provision, patient characteristics and behavior, and health outcomes.

## Limitations

Two key limitations of this approach is that our MICE imputation model is time-invariant, and that it does not account for spatial autocorrelation among nearby clinics. In the future, we may adapt approaches for imputing panel data to suit this task. There are also variables for which we have too few observations to impute. Future work may explore methods of predicting these values with little to no ground truth data.

## Data Availability

The de-identified datasets used and/or analyzed in this current study are available from the corresponding author on reasonable request.

## Appendix

See Tables 2 and 3

**Table 2:** NIHF prediction classifier performance

	Yes	No
Yes	791	780
No	333	19,608
Accuracy	0.948	
Precision	0.504	
Recall	0.704	
F1	0.587	

**Table 3:** Predictive importance of key variables

Variable	Generalized cross-validation (GCV) estimate of error
Age at fist pregnancy	100
Body Mass Index (BMI)	69.9913
Viral load	53.1357
Age at first pregnancy	29.11793
Years of school	28.06922
Children under 18 months: 1	6.261627
Travel time to clinic: 30–60 min	5.991079
WHO weight loss stage: 3	5.981254
Place of delivery 2	5.827017
Pregnancy outcome: unknown/not documented	5.822529
WHO weight loss stage2	5.179188
Visits urban clinic	5.064835
On ARV	5.025331
Travel time to clinic: 1–2 h	4.986507
Delivery assistance	4.309087
Place of delivery 4	4.232822
Delivery assistance 4	3.88988
Travel time to clinic: > 2 h	3.462557
WHO weight loss stage: 4	2.662623
Delivery assistance 5	1.985101

## Abbreviations

EHR: Electronic health records
AMPATH: Academic Model Providing Access to Healthcare
MICE: Multiple imputation by chained equations
NHIF: National Hospital Insurance Fund
